# The Impact of an Emergency Department Front-End Redesign on Patient-Reported Satisfaction Survey Results

**DOI:** 10.5811/westjem.2017.7.33664

**Published:** 2017-09-22

**Authors:** Michael D. Repplinger, Shashank Ravi, Andrew W. Lee, James E. Svenson, Brian Sharp, Matt Bauer, Azita G. Hamedani

**Affiliations:** * University of Wisconsin, Madison, Berbee Walsh Department of Emergency Medicine, Madison, Wisconsin; † University of Wisconsin, Madison, Department of Radiology, Madison, Wisconsin

## Abstract

**Introduction:**

For emergency department (ED) patients, delays in care are associated with decreased satisfaction. Our department focused on implementing a front-end vertical patient flow model aimed to decrease delays in care, especially care initiation. The physical space for this new model was termed the Flexible Care Area (FCA). The purpose of this study was to quantify the impact of this intervention on patient satisfaction.

**Methods:**

We conducted a retrospective study of patients discharged from our academic ED over a one-year period (7/1/2013–6/30/2014). Of the 34,083 patients discharged during that period, 14,075 were sent a Press-Ganey survey and 2,358 (16.8%) returned the survey. We subsequently compared these survey responses with clinical information available through our electronic health record (EHR). Responses from the Press-Ganey surveys were dichotomized as being “Very Good” (VG, the highest rating) or “Other” (for all other ratings). Data abstracted from the EHR included demographic information (age, gender) and operational information (e.g. – emergency severity index, length of stay, whether care was delivered entirely in the FCA, utilization of labs or radiology testing, or administration of opioid pain medications). We used Fisher’s exact test to calculate statistical differences in proportions, while the Mantel-Haenszel method was used to report odds ratios.

**Results:**

Of the returned surveys, 62% rated overall care for the visit as VG. However, fewer patients reported their care as VG if they were seen in FCA (53.4% versus 63.2%, p=0.027). Patients seen in FCA were less likely to have advanced imaging performed (12% versus 23.8%, p=0.001) or labs drawn (24.8% vs. 59.1%, p=0.001). Length of stay (FCA mean 159 ±103.5 minutes versus non-FCA 223 ±117 minutes) and acuity were lower for FCA patients than non-FCA patients (p=0.001). There was no statistically significant difference between patient-reported ratings of physicians or nurses when comparing patients seen in FCA vs. those not seen in FCA.

**Conclusion:**

Patients seen through the FCA reported a lower overall rating of care compared to patients not seen in the FCA. This occurred despite a shorter overall length of stay for these patients, suggesting that other factors have a meaningful impact on patient satisfaction.

## INTRODUCTION

Emergency department (ED) volumes in the United States continue to grow, leading to crowding, delays in care, and increased wait times.^1^ To address this growing crisis, EDs have used innovative methods, including initiatives such as physician triage, observation units, and fast-track areas.^2^ A number of departments have used a model in the ED that focuses on “vertical patient flow,” in which patients can be evaluated, managed, and rendered a disposition in the setting of the waiting room and without occupying a traditional ED bed.^3^ Outside of the ED, other targets have included downstream processes such as facilitating hospital discharges and smoothing elective surgical scheduling.

At our ED, a Level I adult and pediatric trauma and burn center with an emergency medicine residency program, we have implemented a front-end redesign, which focuses on vertical patient flow as a means to minimize delays in care, particularly care initiation.

The physical space for this new flow paradigm was a sectioned-off part of the waiting room called the “Flexible Care Area” (FCA) and consisted of three rooms with an adjoining work area for the care team. This was staffed by an emergency physician, a nurse, and an emergency medical technician. The guiding principle for the staff in this area was to ensure a safe waiting room (i.e., briefly evaluate each patient to ensure no time-sensitive conditions were being missed), while also addressing bottlenecks in the diagnostic process; for example, laboratory tests, imaging tests, and even specialist consultation can be ordered from this area. Additionally, lower-acuity patients without the need for diagnostic tests or consultation, or whose diagnostic tests could be resulted rapidly, could have their entire episode of care completed by the FCA staff.

The explicit prioritization for this flow model, however, was to first address higher-acuity patients and expedite their workup prior to tackling the needs of lower-acuity patients. The team staffing the FCA was responsible for identifying patients who would benefit from evaluation and treatment in the FCA, diverting these patients from the waiting room to initiate their care. Previously, we have shown that using this flow model decreases overall length of stay (LOS) for all ED patients with emergency severity index (ESI) levels 3 and 4.^3^ These data are in line with national trends showing that front-end changes, such as fast track and vertical flow, have decreased delays in care.^2^,^4^

In an ever-evolving patient-centered ED model, however, it is not enough to measure improvements simply by time-related metrics. The Centers for Medicare and Medicaid Services use patient satisfaction as a key marker of value. Since LOS and time to initiation of care are fundamental determinants of patient satisfaction with ED care,^5^ we hypothesized that implementation of our FCA would lead to greater patient satisfaction for those seen in the FCA. Therefore, the purpose of this study was to quantify the impact of our FCA model on patient satisfaction by comparing the Press-Ganey scores of discharged patients seen entirely in FCA vs. those cared for primarily in the main ED, even if their care was begun in the FCA. Secondarily, we aimed to demonstrate the impact of individual components of care, including diagnostic and therapeutic interventions, on overall patient satisfaction.

**Population Health Research Capsule**

What do we already know about this issue?

ED length of stay (LOS) is viewed as a key driver of patient satisfaction. Interventions aimed at decreasing LOS theoretically improve satisfaction.

What was the research question?

Can an ED redesign that embraces vertical patient flow decrease LOS and increase satisfaction?

What was the major finding of the study?

Though vertical patient flow decreases ED LOS, it was not observed to impact patient satisfaction.

How does this improve population health?

Future studies of drivers of ED patient satisfaction should investigate factors beyond time metrics, possibly more focused on the physician-patient interaction.

## METHODS

### Study Design and Setting

This was a retrospective study of patients discharged from our ED from 7/1/2013 to 6/30/2014. The study was conducted at an academic, tertiary-care hospital with approximately 50,000 ED patient visits annually during the study period. This study was HIPAA-compliant and deemed exempt from review by our institutional review board.

### Selection of Participants

We included in this study discharged patients who were seen in our ED during the study period and returned a Press-Ganey survey. Per hospital protocol, Press-Ganey surveys are sent one week after an ED visit to a representative sample of patients (every third adult patient and every other pediatric patient) discharged from the ED. Patient selection for survey participation was not part of this study.

### Methods and Measurements

We reviewed the EHRs of included patients, including clinical documentation and bed tracking, to ascertain whether their entire episode of care occurred in the FCA (termed “FCA patients” for the purposes of this study). All other patients were considered “non-FCA” patients, including the cohort of patients who had their care initiated in the FCA area, but were subsequently transitioned to complete their care in the main ED. The FCA was open 4pm–12am daily and though many patients had their care initiated in this setting, less than 10% had it completed there.

The primary data abstractor was a research assistant who was trained on a standardized data abstraction protocol by the principal investigator (PI). After abstracting 25 charts together, the PI also re-abstracted data from another 100 charts to verify data integrity. No significant discrepancies were identified during this process. Abstracted data included age, imaging tests performed, opioid pain medication received, laboratory tests ordered, whether the patient had all care rendered in the FCA area, ED LOS, time to being placed in a room, time from being placed in a room to seeing a physician, and ESI score. We tabulated all data in a Microsoft Excel 2013 spreadsheet (Microsoft Corporation, Redmond, WA).

### Outcome Measures

We used Press-Ganey patient satisfaction surveys to evaluate patient’s perceptions of care. Responses on survey items were reported on a five-point scale (Very Poor, Poor, Fair, Good, Very Good) but for the purpose of our primary analysis were dichotomized into “Very Good” (VG) and “Other,” where “Other” represented all other possible ratings. The rationale for the dichotomization was that our institution, as well as many others, only use the top rating percentage when assessing system and provider performance. Our primary outcome was the response to the survey item related to “Overall rating of care received during your visit.” Ratings were also abstracted for responses related to perceived quality of physician and nursing care. These questions included the following: “Courtesy of the doctors who cared for you;” “Degree to which these doctors took the time to listen to you;” “Concern these doctors showed to keep you informed about your treatment;” and “Concern these doctors showed for your comfort while treating you.” A similar set of questions was asked about perceived quality of nursing care. As with the overall rating of care, we dichotimized the answers to these questions based on whether the response was VG or anything else.

### Data Analysis

Our primary analysis was comparing the percentage of FCA patients who rated their overall care as VG vs. the percentage of all other patients who rated their care as VG, though we do also report the mean score for FCA vs. non-FCA patients by simply converting the survey responses to an ordinal scale (1–5). Secondarily, we performed univariate and multivariate regression analysis for each of the seven variables abstracted in chart review to ascertain the correlation between each variable and reporting of care as VG (reported as odds ratios [OR]). We also calculated the percentage of patients who rated their physician and nurse as VG in several domains; we again performed a regression analysis and report the OR for rating their overall care as VG based on each survey item in this set. We did not include missing survey responses in data analysis. We used Fisher’s exact test to calculate statistical differences in proportions, while the Mantel-Haenszel method was used to calculate OR during regression analyses. We conducted statistical analysis using Statistical Analysis System (SAS) version 9.4 (SAS Institute Inc., Cary, NC).

## RESULTS

### Study Population

During the study period, 50,358 patients were seen in our ED. Of these, 34,083 were discharged, 14,075 of whom were sent a Press-Ganey survey to complete. A total of 2,358 patients (16.8%) completed and returned the survey, which comprises our study sample. Of these, 133 patients (5.6%) had their entire episode of care conducted in the FCA only. Assuming an estimated 10 patients completed their care in FCA daily, then data from approximately 3.65% (133/3,650) of FCA patients are included in this analysis. The mean age of patients who returned a Press-Ganey survey was 47.9 (standard deviation 23.8) years. Table 1 displays characteristics of all patients in the study cohort and compares the characteristics of patients who rated their overall care as VG vs. those who did not.

### Main Results

Across all patients in the study, 62.6% (1,388/2,218) rated their overall care for the visit as VG. Of FCA patients, 53.4% (71/133) reported their care as VG, compared with 63.2% (1,317/2,085) of discharged patients cared for in the main ED (p=0.027). The mean survey rating for patients seen in FCA was 4.26 (95% confidence interval [CI] [4.09–4.43]) while it was 4.41 (95% CI [4.37–4.45]) for non-FCA patients; the p-value for difference in scores was non-significant (p=0.09).

### Secondary Analyses

#### Comparison of FCA Vs. Other Patients

We profiled the patients based on whether their entire care episode took place in the FCA vs. those who were seen primarily in the main ED, even if their care was begun in the FCA (Table 2). A smaller proportion of patients seen exclusively in the FCA had advanced imaging performed (12% versus 23.8%, p=0.001). This result was also seen in laboratory tests done (24.8% versus 59.1%, p=0.001). LOS (159.0 ±103.5 min versus 223.0 ±117.0 min, p=0.001) and acuity (median ESI 4 versus ESI 3, p=0.001) were lower for FCA patients than non-FCA patients. Patient age and time from being placed in a room to seeing a physician were not statistically different for these groups. Of note, time from being registered to being placed in a room was statistically higher for FCA patients compared to non-FCA patients (23.1 versus 10 minutes, p=0.001).

#### Effects of Physician and Nursing Care on Patient Satisfaction Score

There was no difference in physician and nurse ratings comparing those patients seen in FCA vs. those not seen in FCA. However, patients who reported physician care as VG were more likely to rate their overall care in the ED as VG, with OR ranging 21.8–23.5 (Table 3). Nursing care, however, had minimal or no impact on overall satisfaction, with OR ranging 1.11–1.23. Thus, while we found no statistical difference in survey results for questions concerning physician and nursing care between FCA and non-FCA patients, we did find a highly significant increased odds of overall patient satisfaction if respondents had a high opinion of physician care, but not of nursing care.

#### Univariate Logistic Regression

Patients were less likely to rate their overall care as VG if they were seen exclusively in the FCA (OR 0.67, 95% CI [0.47–0.95]). They were also less likely to rate their overall care as VG if they had opioid pain medicines administered (OR 0.73, 95% CI [0.6–0.9]). Further, the percentage of patients reporting their care as VG was not affected by having imaging tests performed (either radiograph or advanced imaging), having laboratory tests performed, or being signed out to another team (p-values ranging 0.08 to 0.86). See Table 4 for a complete listing of OR.

#### Multivariate Logistic Regression

When modeling the effect of all individual elements extracted from EHR review, being cared for in the FCA (OR 0.57, 95% CI [0.39–0.83], p=0.004) and increasing age (OR 0.99, 95% CI [0.986–0.993], p<0.001) were the two parameters associated with decreased overall patient satisfaction with care. Shorter LOS was statistically positively, but practically very weakly, associated with satisfaction (OR 1.004, 95% CI [1.003–1.005], p<0.001). All other variables did not affect patient satisfaction to a statistically significant level.

## DISCUSSION

Responses on patient satisfaction surveys have been identified as a key marker of value in the patient care experience. In this retrospective evaluation of patient satisfaction with overall care in the ED, we found that operationalizing a vertical patient flow model, which we termed and housed in the Flexible Care Area, did not yield an improvement in the percentage of patients who rated their overall care as “Very Good.” However, we were able to demonstrate that the primary objective of this new patient care paradigm was achieved: overall LOS was, in fact, shorter for patients seen in the FCA (159.0 ±103.5 min versus 223.0 ±117.0 min). It seems that while improving time-related metrics may decrease overall ED crowding, this may not equate to an improved patient experience.

Focusing on LOS alone as the primary driver of patient satisfaction appears to have missed the mark and is an oversimplification of what drives patients’ needs from an ED visit. Though it is a relatively easy measure to trend, further study into the drivers of patient experience will surely show that many other factors are as important. For example, a significant, unexpected result through secondary analysis of the survey data suggests that while patient satisfaction with physician care is highly correlated with overall care rating, patient satisfaction with nursing care had minimal or no effect on overall patient satisfaction.

To uncover possible differences in measurable components of care for patients seen solely in FCA compared with all others, we assessed a number of interventions for the two groups. Though we did observe a difference in the number of interventions performed for FCA versus non-FCA patients, we found no difference in the odds of reporting satisfaction with overall care as VG if patients had these interventions performed. The one exception to this finding was if a patient received opioid pain medicines (whether in FCA or not) – almost paradoxically, those who did were less likely to report their care as VG, a finding that has been reported previously.^6^ This may be confounded by the fact that FCA patients were both less likely to receive these pain medicines and less likely to report their overall care as VG. Informal querying of nursing staff in the FCA suggests the reason for decreased use of opioid pain medicines was two-fold. First, patients did not remain in the physical FCA area for the entirety of their stay – in the spirit of embracing the vertical patient flow philosophy, they were asked to return to the waiting room and therefore were not as likely to have their pain level assessed. Additionally, FCA nursing staff was reticent to administer opioid pain medicine in the FCA because of concerns of these patients later being unmonitored in the waiting room.

Prior studies have compared overall patient satisfaction after implementation of a fast-track area, demonstrating that overall satisfaction increases.^7^ However, our FCA is different than a traditional fast-track system; prioritization was given to first address higher-acuity patients and expedite their workup prior to tackling the needs of lower-acuity patients, who are the usual target of fast-track systems. Despite this broader mission to decrease time to care initiation, our results demonstrated a different finding than that previously reported with fast tracks: patients seen in the FCA rated their overall care less favorably. This was in spite of overall LOS being shorter for those patients seen exclusively in FCA. Though such patients had lower ESI scores than the general ED population, they likely had to wait longer than traditional fast-track patients since higher-acuity patients, destined for the main ED, were prioritized to have their care initiated in the FCA before the lower acuity, fast track-type, patients. Additionally, those general ED patients who were initially seen in FCA benefited from decreased care delays afforded by FCA, but were not included in the FCA group for analysis purposes in this paper. These two departures from usual fast track-type systems may help to explain the difference in findings.

One of the factors that may negatively impact patient satisfaction is the inherent nature of a vertical patient flow setting like our FCA; many patients are not given their own private room for the duration of their stay. FCA patients are frequently brought to and from FCA rooms, and asked to return to the waiting room while their diagnostic studies are pending. These patients, once returned to the waiting room to await test results, are not given many of the amenities that a patient in a regular ED room is given while waiting, including private TVs and access to a nursing call button to help address immediate needs. Our findings are similar to previous studies, which have demonstrated that ED hallway-bed usage is associated with lower ED satisfaction and lower satisfaction with the overall hospitalization.^8^ Similarly, because patients are not seen and treated in the traditional manner, many patients seen in FCA may have the perception that they are not receiving the same care as a patient seen in a traditional room.

It is important to note that patients’ perceptions of care provided by the physician was not different when comparing the FCA group and non-FCA group. This is not surprising given that emergency physicians who staff the FCA are the same physicians who work in the main ED. Yet, there seems to be a disconnect between patients’ perceived medical care and actual care. Several studies have shown that increased wait times and time to see a doctor can lead to adverse outcomes, including prolonged time to antibiotics in severe pneumonia^9^ and time to thrombolytics in acute myocardial ischemia.^10^ Studies have shown that patients seen in a fast-track setting are under-evaluated and undertreated for pain,^11^ though we demonstrated no statistically significant difference in the percentage of patients receiving opioid pain medications in FCA versus the main ED. However, patients who received opioid pain medication were less likely to report their overall care as VG with an OR of 0.73 (95% CI [0.6–0.9]).

## LIMITATIONS

This study has several limitations. First, this is a single-center, retrospective study of a relatively small patient population at an academic medical center, which limits the study’s power and generalizability. (For example, compared with community hospitals, academic medical centers generally have longer baseline LOS; and the FCA was only open for eight hours a day [4pm–midnight]). The fact that it was retrospective, however, limits the potential for the Hawthorne effect and other forms of information bias. Second, only cursory demographic information was used to control for confounding in this study. It may be that there was a greater proportion of patients seen in FCA with chronic complaints, or complaints that result in an overall lower patient satisfaction score, no matter the setting in which they are seen. However, the same providers who staffed the FCA also staffed the main ED, which should limit confounding by provider type. Though we considered using each provider as his/her control as another potential confounder, it would be nearly impossible to disentangle the effect of a resident from that of the attending or if the care spanned more than one shift/physician team.

Also, patients who benefited from the FCA’s early initiation of care but completed their care in the main ED would have been categorized as “non-FCA” by our study definitions. This fact likely diminished the observed impact of FCA on patient satisfaction. However, we also noted that throughput demands at times caused even some ESI level 2 patients (2.3% of the total cohort) to have their care completed in the FCA. Though the overall impact of FCA on LOS and patient satisfaction scores for those not seen in FCA was not part of this study, we previously reported that the implementation of the FCA did lead to decreased LOS for those patients.^3^

Further, use of Press-Ganey scores is dependent on the response rate, and patients in FCA who rated their overall care as VG may have responded to the survey at a lower rate than those patients seen in the main ED. The overall response rate was also low for a survey study, though in line with previously reported Press-Ganey response rates,^12^ which limits our ability to make valid conclusions regarding respondents’ opinions. However, given the penetrance of the Press-Ganey survey into the hospital patient-satisfaction industry, and despite its low overall response rate, we felt that its use for this study was acceptable.

## CONCLUSION

Patients who had their entire episode of care conducted through our ED’s front-end vertical patient flow redesign area reported a lower rating of overall care when compared to all other patients, despite a shorter overall length of stay. Clearly, factors beyond length of stay have a meaningful impact on patient satisfaction and must be taken into consideration as EDs work to balance throughput with patient satisfaction. As hospitals continue to optimize ED throughput,^13^ it will be important to also evaluate how these unmet patient expectations contribute to patient satisfaction. Policymakers should also take note that patient experience can be negatively impacted by interventions aimed only at throughput metrics.

## Figures and Tables

**Table 1:** Characteristics of study patients who responded to Press-Ganey survey Results are reported for all patients as well as the subgroup who reported their overall care as “Very Good,” or “Other,” which included “Very Poor,” “Poor,” “Fair,” and “Good.” For each variable, the mean and standard deviations are reported.

Variable	Very good (n= 1437)	Other (n=877)	p-value	Overall
Age (years), SD	49.4 ±23.7	45.3 ±22.5	<0.001	47 ±23.8
Length of stay (minutes), SD	206.9 ±107.4	246.3 ±130.5	<0.001	222.6 ±118
Time to room (minutes), SD	7.8 ±21.2	19.0 ±35.40	<0.001	12.2 ±28
Time from room to first physician (minutes), SD	12.5 ±13.9	16.9 ±19.5	<0.001	14.3 ±16.4
Acuity (ESI score)			0.001	
1	0.1%	0.0%		0.04%
2	19.3%	12.9%		16%
3	61.5%	66.4%		61.2%
4	18.6%	19.3%		18.1%
5	0.6%	1.3%		0.8%

*ESI*, emergency severity index; *SD*, standard deviation.

**Table 2:** Comparison of characteristics for patients seen in the Flexible Care Area (FCA) vs. non-FCA. The percentage of patients who had one of four different interventions are reported here as is the overall emergency department (ED) length of stay; time from ED arrival to being placed in a room; time from being placed in a room to seeing a physician; age; and triage acuity score.

Variable	FCA (n=133)	Non-FCA (n=2125)	p-value
Patient received a radiograph	48.1%	56.0%	0.08
Patient received an advanced imaging study	12.0%	23.8%	0.001
Patient received any opioid pain medication	18.8%	22.9%	0.33
Patient had laboratory testing performed	24.8%	59.1%	0.001
Length of stay (minutes)	159.0 ±103.5	223.0 ±117.0	0.001
Time to room (minutes)	23.1 ±33.7	10.0 ±24.9	0.001
Time from room to first physician (minutes)	11.9 ±9.1	14.1 ±15.7	0.65
Age (years)	48.5 ±18.4	47.6 ±27.3	0.68
Acuity (ESI score)			0.001
1	0.0%	0.1%	
2	2.3%	17.9%	
3	40.0%	64.7%	
4	53.9%	16.7%	
5	3.9%	0.7%	

*ESI*, emergency severity index.

**Table 3:** Ratings of physician and nurse care. Percentage of patients who rated their physician and nurse care as “Very Good” are shown for patients seen in the Flexible Care Area (FCA) vs. those not seen in the FCA. The reported p-value tests the difference between FCA and non-FCA patients reporting “Very Good” to each question. The last two columns report the odds (and confidence interval) of rating overall care as “Very Good” when the patient also reported “Very Good” for each statement.

Individual questions regarding provider care	FCA	Non-FCA	p-value	OR	95% CI
Doctors kept me informed about treatment	64.9%	62.1%	0.57	21.8	17.5–27.3
Doctors were courteous to me	66.2%	70.1%	0.38	22.03	17.4–27.8
Doctors took the time to listen to me	63.6%	65.9%	0.64	22.4	17.9–28.1
Doctors were concerned about my privacy	62.1%	63.5%	0.78	23.5	18.8–29.4
Nurses kept me informed about treatment	58.9%	65.7%	0.12	1.16	0.97–1.39
Nurses were courteous to me	67.7%	74.9%	0.08	1.23	1.02–1.5
Nurses took the time to listen to me	67.4%	71.2%	0.36	1.11	0.92–1.34
Nurses were concerned about my privacy	61.7%	70.1%	0.05	1.18	0.98–1.42
Nurses were attentive	64.3%	69.8%	0.2	1.14	0.95–1.37

*CI*, confidence interval; *OR*, odds ratio.

**Table 4:** Odds ratios for various interventions. Odds of reporting overall care as “Very Good” when evaluated for each individual potential determinant of perceived care. Laboratory testing included any test (blood, urine, etc) that was sent to the hospital laboratory.

Variable	OR	95% CI
Patient signed out to another team	0.8	0.62–1.04
Had an X-ray performed	0.98	0.83–1.16
Had a CT or MRI performed	0.91	0.75–1.12
Received opioid pain medicines	0.73	0.6–0.9
Received intravenous opioid pain medicines	0.81	0.65–1.02
Had laboratory testing performed	1.01	0.86–1.2
Seen in the FCA only	0.67	0.47–0.95

*CI*, confidence interval; *CT*, computed tomography; *FCA*, flexible care area; *MRI*, magnetic resonance imaging; *OR*, odds ratio.
